# The European Union Committee of Experts on Rare Diseases: three productive years at the service of the rare disease community

**DOI:** 10.1186/1750-1172-9-30

**Published:** 2014-02-28

**Authors:** Ségolène Aymé, Charlotte Rodwell

**Affiliations:** 1 INSERM, US14 - Orphanet, Paris, France

**Keywords:** European policy, Recommendations, National plans/strategies for rare diseases, Centres of expertise, Access to orphan medicinal products, Patient registries, European reference networks

## Abstract

The European Union Committee of Experts on Rare Diseases was entrusted with aiding the European Commission in a number of tasks, ranging from the monitoring of initiatives, to recommending improvements and actions to be pursued in the future, in addition to helping strengthen liaison at both European and International levels in the field of rare diseases. The three-year mandate of the EUCERD drew to a close in July 2013 with an impressive record. The EUCERD has laid down the foundations for future work so as to continue to advance in the key areas that have been identified as of interest for the rare disease community at large: centres of expertise, European Reference Networks, patient registries and databases, newborn screening, and indicators for national rare disease plans/strategies. The work of the Committee should now be continued by the newly formed European Commission Expert Group on Rare Diseases.

## Introduction

**Figure 1:**
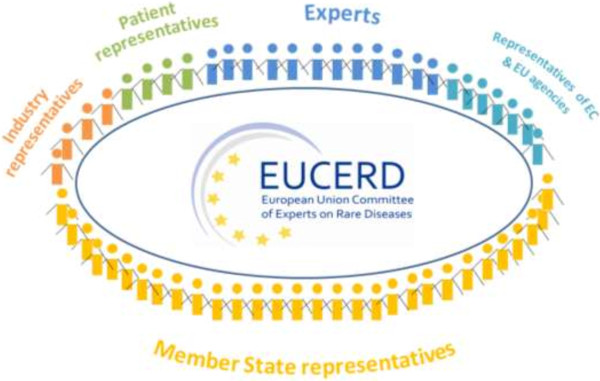
Composition of the EUCERD.

The three-year mandate of the European Union Committee of Experts on Rare Diseases [1] drew to a close in July 2013 with an impressive record. These results were achieved thanks to the unique nature of the forum it provided for the discussion of key topics for the rare disease community, bringing together representatives from all 28 European Member States, experts, patient representatives and members of the Industry, as well as the European Commission [Figure 1]. The enthusiastic participation of the 51 members of the EUCERD allowed the EUCERD to foster exchanges of relevant experience, policies and practices in the field of rare diseases, and help the European Commission and the Member States prepare and implement activities in the in the field of rare diseases.

**Figure 2:**
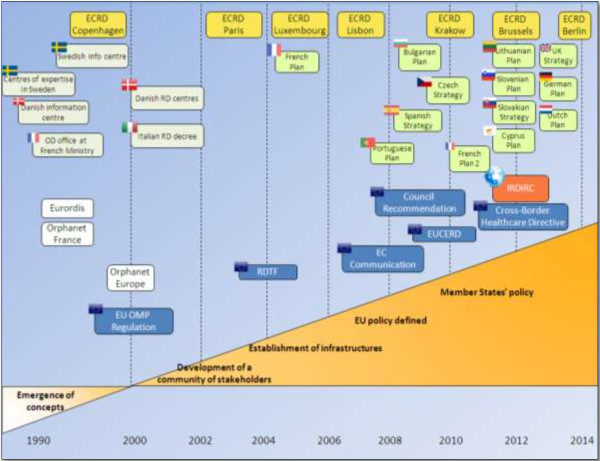
Evolution of concepts and initiatives in the field of rare diseases in Europe.

Established via the European Commission Decision of 30 November 2009 (2009/872/EC) [2], the EUCERD responded quickly to the evolving needs of the rare disease community [Figure 2] following the *Commission Communication Rare Diseases: Europe’s Challenge* (2008) [3] and the *Council Recommendation on an Action in the Field of Rare Diseases* (2009) [4]. These texts defined a European policy in the field and notably encouraged Member States to elaborate national plans/strategies for rare diseases by the end of 2013 to *“[guide] and [structure] relevant actions in the field of rare diseases within the framework of their health and social systems”*. Although other non-European countries and world regions have policies in place relative to orphan medicinal products, Europe is the only region with a public health policy in the field of rare diseases.

**Figure 3:**
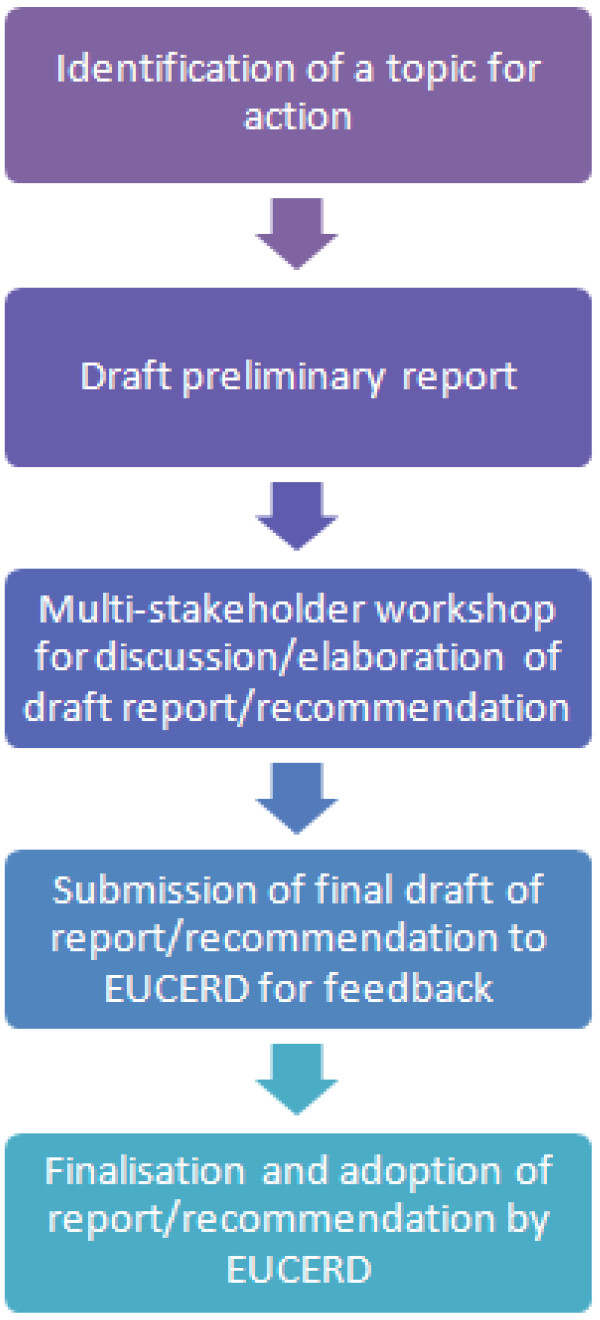
The EUCERD’s work process.

The EUCERD selected from the key areas cited by the Commission Communication and Council Recommendation a number of topics which had to be tackled immediately in order to clarify concepts and guide the reflection underway at national level. Most of these selected topics had already been tackled by the European Commission’s Rare Diseases Task Force (RDTF), the EUCERD’s predecessor, and there was sufficient consensus to elaborate sets of recommendations for the European Commission and Member States. Supported via two consecutive Joint Actions between Member States (N°2008 22 91 and N°2011 22 01), the EUCERD thus organised a number of expert workshops over the years to elaborate the recommendations in areas concerning the organisation of expert services (centres of expertise and European Reference Networks), data collection, and access to medicines. For each topic the process started with the identification of the scope of the issue and a fact-finding mission concerning the state of the art of the topic. This information was used to produce a preliminary report, or a draft recommendation, which provided material in turn for an expert workshop to discuss and elaborate a recommendation which was then submitted for discussion and adoption by the EUCERD [Figure 3]. The Recommendations have been disseminated by the members of the Committee and through OrphaNews Europe [5], the newsletter of the committee with 14 000 registered readers.

### Centres of expertise : the key stone of national plans and strategies for rare diseases and a step towards European networks

The first area of the *Council Recommendation* to be dealt with by the Committee concerned the centres of expertise for rare diseases and European Reference Networks for rare diseases. The identification, and creation, of centres of expertise for rare disease is a key element of the *Council Recommendation* and central to national rare disease plans/strategies. There are around 6 000 rare diseases and most are unknown to healthcare professionals so rare diseases patients suffer from not knowing where to consult. To overcome this, some Member States have established centres specialised in some rare diseases/groups of rare diseases which have proven to be very efficient in providing quality of care for patients. The networking of these centres could lead to the gathering of the scarce expertise concerning these diseases at European level, in order to ensure equal access to accurate information, appropriate and timely diagnosis and high quality care for rare disease patients.

This area had been previously identified by the former RDTF as a priority topic with a dedicated working group, as networks of centres of expertise had been defined by the High Level Working Group on European Centres of Expertise as an area of European added-value with consensus on the benefits of a cross-border approach [6]. Indeed, the RDTF had previously established a list of criteria for designation of centres of expertise which served as the starting point for the EUCERD’s reflection on this subject. Currently, the organisation of centres of expertise varies greatly from country to country: few countries currently have a designation process in place, and the designation criteria vary across these countries, and sometimes even from region to region. By revisiting the work of the RDTF, the EUCERD was able to draft and adopt recommendations relevant to the current situation concerning the definition of the missions, scope and criteria for designation and evaluation of centres of expertise. The EUCERD’s *Quality Criteria for Centres of Expertise for Rare Diseases in Member States* (2011) [7] have been very well received in the EU Member States and are being actively used as a starting point in the elaboration of the actions concerning the organisation of care in their national plans/strategies. Member States have appreciated the clarification of concepts provided by the Recommendations which can be tailored to the countries’ specificities. It is hoped that the use of these Recommendations will help to harmonise the criteria for designation of centres from country to country, in preparation for the establishment of the future European Reference Networks for rare diseases, for which centres of expertise are the building blocks [Table 1].

**Table 1:** Key facts and state of play in European countries in December 2013: Centres of expertise for rare diseases (CE RD)^1^


•	1 European country with designated CE RD in the scope of a national plan for rare diseases
•	5 European countries with officially designated CE RD
•	15 European countries with non-designated CE RD acknowledged by health authorities to varying degrees
•	9 European countries with CE RD recognised by reputation only
•	16 European countries with plans to designate CE RD in their national plans/strategies for RD
•	*EUCERD Recommendations on quality criteria for centres of expertise for rare diseases in Member States* adopted on 24 October 2011
•	Consensus on 45 recommendations covering the mission and scope, criteria for designation, process of designation and evaluation, and European dimension of CE RD

^1^Data as of December 2013, reproduced from S. Aymé, C. Rodwell (eds.), **2014 Report on the State of the Art of Rare Disease Activities in Europe,** to be published in July 2014.

### European Reference Networks for rare diseases: the European dimension of health care pathways

Collaboration of centres of expertise through European Reference Networks (ERNs) is highlighted in the *Council Recommendation*, whilst the creation of such European structures is a key element of the *Directive on the application of patients’ rights in cross-border healthcare (2011/24/EU)* (9 March 2011) [8], which specifically cites rare diseases as an area of interest for ERNs. The concept of ERNs for rare diseases had been developed over a number of years before the creation of the EUCERD by a dedicated RDTF working group mandated by the HLG. This working group ascertained that the treatment of rare diseases demands multidisciplinary care which is sometimes not available at local or national level and worked on establishing a set of recommendations concerning ERNs [9]. The EUCERD decided, once the concept of centres of expertise was well established, to take stock of this work in the context of the Directive in order to establish a recommendation for both the European Commission and the Member States. The *Recommendations on European Reference Networks for Rare Diseases* (2013) [10] highlights the specificities of rare diseases to be taken into account when considering the scope, mission, governance, designation and evaluation of such ERNs in the future activities planned around the Cross-Border Healthcare Directive. The reflections during the elaboration of the document have helped develop the concept of ERNs in the field of rare diseases. The adopted EUCERD Recommendations have provided guidance to Member States in process of elaborating the healthcare pathways at both the national and European levels in the scope of their national plans/strategies for rare diseases to ensure that this future possibility is envisaged from their inception. The Recommendations have also now fed into the process underway at the European Commission to prepare the legal framework for the implementation of the future ERNs, of which a number, it is hoped, will be dedicated to rare diseases. Stakeholders are now waiting for confirmation of how the sustainability and designation of these ERNs will be organised at European level. This question has been partially answered by the mention of support for projects concerning the modelling and validation of system methodologies for European Reference Networks in the Horizon 2020 call [11] [Table 2].

**Table 2:** Key facts : European Reference Networks (ERNs) for rare diseases


•	Rare diseases cited in Directive on the application of patients’ rights in cross border healthcare (2011/24/EU) (9 March 2011) as priority area for ERNs
•	*EUCERD Recommendations on European Reference Networks for Rare Diseases* adopted on 31 January 2013
•	Consensus on 21 recommendations covering mission, vision and scope, governance, composition, funding and evaluation, and designation of ERNs for Rare Diseases

### Patient registration and data collection: Gathering information at European level on rare diseases

Patient registries are a key aspect of national plans/strategies for rare diseases and are cited as a crucial source of information on rare diseases, in terms of basic and clinical research as well for epidemiological and public health purposes, to be supported at national and European level in the *Council Recommendation*. Patient registries are a key tool for gathering the scarce knowledge relevant to rare diseases so as to improve the understanding of these conditions and the treatment available to patients, as well as the planning of healthcare services for these diseases. Over 640 rare disease registries exist in Europe according to data extracted from Orphanet [12] in December 2013, with the majority concerning diseases of groups of diseases for which there is an innovative treatment either in development or already on the market. At the national level many countries are considering in the scope of their national plans/strategies the best way to collect data relative to rare disease patients, and at the Community level the European Commission is in the process of establishing a European Platform for Rare Disease Registration. The EUCERD thus decided to build on the previous work of the RDTF on patient registries to elaborate a set of *Recommendations on Rare Disease Patient Registration and Data Collection*[13] with the aim of setting down the consensus reached to date and to guide all stakeholders at this crucial moment in the collective reflection on the topic.

The EUCERD’s recommendations provide the consensus to date in the field and call for the involvement of all stakeholders in the designing, maintenance and governance of registries in the future, as well as public-private partnerships for long-term sustainability. These recommendations will serve all stakeholders in the field, including Member States establishing their national plans/strategies for rare diseases, the European Commission services in their reflection on the sustainability of registries for rare diseases at European level, and members of the Industry looking to fulfil their post-market authorisation obligations. These recommendations are also feeding into discussions on data collection and registration underway at international level in the working groups of the International Rare Disease Research Consortium (IRDiRC) [14] notably concerning interoperability and pooling of data for research purposes. Stakeholders at European and national level are now awaiting more information from the Commission concerning the mission of the European platform [Table 3].

**Table 3:** Key facts and state of play in December 2013: Rare disease patient registration and data collection


•	Around 640 rare disease registries in Europe^1^
•	Majority of registries are academic
•	Some RD have more than one registry, many RD have no registry
•	*EUCERD Core Recommendations on Rare Disease Patient Registration and Data Collection* adopted on 5 June 2013
•	Consensus on 6 main areas : international operability, sources of data, collection of data, good practices, use of data for regulatory purposes, and sustainability

^1^ Data as of December 2013 from **Orphanet** [http://www.orpha.net] concerning European Member States and surrounding European countries in the Orphanet Consortium.

### Steps towards better and timely access to orphan medicinal products for rare diseases : Improving the Clinical Added Value of Orphan Medicinal Products information flow

The EUCERD also worked, at the request of the European Commission following a Tender report on the subject, on the issue of equitable and timely access to approved orphan medicinal products for rare diseases. This matter is highlighted by the *Communication* and *Council Recommendation* where sharing of relevant knowledge is promoted in order to *“minimise delays in access to orphan drugs for rare disease patients*”. Although the European Regulation on Orphan Medicinal Products (141/2000) [15] establishes a centralised procedure for the designation of orphan medicinal products and puts in place incentives for the research, marketing and development of orphan medicinal products, access to these medicines is often difficult and unequal from country to country. In order to advance the reflection on this matter, the EUCERD brought together key stakeholders to discuss these issues and adopted in 2012 a *Recommendation on Improving Informed Decisions Based on the Clinical Added Value of Orphan Medicinal Products Information Flow*[16]*.* The EUCERD’s recommendation highlights potential ways to facilitate scientific information exchange in order to support Member States in their processes of making informed decisions on the scientific assessment of the clinical effectiveness of an orphan medicinal product. The EUCERD recommends creating an information flow between individual Member States and between Member States and EU bodies to bridge existing knowledge gaps, especially at the time of market authorisation. Ultimately this will accelerate access to approved orphan medicinal products by providing the most robust set of information possible, while encouraging pricing and reimbursement decisions based on the value of these products and promoting good medical practices throughout the EU. This recommendation is the fruit of dedicated participation from all stakeholders, and has been warmly welcomed by across the board. The EUCERD has submitted this proposal to the European Commission in order to inform the next steps in the process. Stakeholders now await further details concerning pilot initiatives concerning suggested actions at European level [Table 4].

**Table 4:** Key facts and state of play in December 2013: The clinical added value of Orphan Medicinal Products^1^


•	1234 positive opinions for orphan product designation from 1798 applications submitted since 2000 at EU level and a total of 1219 European Commission designations
•	85 orphan designated products have received marketing authorisation by end of 2013 at EU level
•	*EUCERD Recommendation on Improving Informed Decisions Based on the Clinical Added Value of Orphan Medicinal Products Information Flow* adopted in September 2012
•	Recommendation proposes four key time points for information sharing to improve the pricing and reimbursement decision process

^1^Data from the report of the European Medicines Agency's Committee on Orphan Medicinal Products December 2013 [http://www.ema.europa.eu/docs/en_GB/document_library/Committee_meeting_report/2014/01/WC500159429.pdf].

### Indicators for rare disease national plans/strategies : Monitoring the progress made in national policy

To provide Member States elaborating their national plans/strategies for rare diseases with guidance concerning indicators to help monitor the elaboration and implementation of these plans at European level, the EUCERD has also adopted *Recommendations on core indicators for rare disease national plans/strategies* (2013) [17]. Indicators are vital tools for assessing the outcomes and success of these measures. These indicators were also intended to provide data for the European Commission’s report on the implementation of the *Commission Communication* and *Council Recommendation* and to serve as a basis for indicators at national level. Although each country will have to tailor indicators to suit the measures foreseen in their national plan/strategy, the Recommendations may serve as a starting point for some countries. They will be reviewed in time to reflect the experience of Member States in the monitoring of their plans/strategies.

### Newborn screening: A definition of possible areas of European collaboration

The EUCERD also decided to investigate areas of potential collaboration at European level in the field of newborn screening, at the request of the European Commission following a Tender report on the subject [18]. Currently, a great heterogeneity of practices can be observed from country to country in the EU and there is no systematic approach to this topic at EU level : this considered, the EUCERD took its cue from the *Council Recommendation* encourages that Member States “*develop European guidelines […] population screening, while respecting national decisions and competences”*. After examining the results of the Tender study, the Committee was able to identify a number of possible topics for collaboration which respected the principle of subsidarity and after discussion on the subject at Committee level an Opinion [19] was adopted in July 2013. It was decided to not prioritise these elements, which were submitted as an Opinion to the European Commission, the Member States and third parties for further consideration. This document is an important first step in this complicated area.

### The EUCERD State of the Art Report: Monitoring progress and reporting on initiatives

The EUCERD has played an important role in the monitoring and dissemination of the results of measures taken at Community and national level in the field of rare diseases, both through the annual report on the *State of the Art of Rare Disease Activities in Europe*[20] and the bi-monthly newsletter of the EUCERD *OrphaNews Europe*[5]*.* The *State of the Art* report is a comprehensive document updated each year which presents the current state of activities at both European and Member State level in a range of different areas of the rare disease field, such as rare disease national plans/strategies, expert services, patient organisations, research and orphan medicinal product policy. The first volume of the report presents an overview of the field which presents to the general public in an easily accessible manner the achievements to date and challenges for the future, whereas the following volumes present the environment in greater detail. The report is also available in country-specific volumes for reuse at national level to inform stakeholders of the state of play.

Both the report and the newsletter are supported by the EUCERD Joint Action and entrusted to the Scientific Secretariat of the EUCERD, with valuable contributions and input from members of the Committee. Notably, the information collected for the *OrphaNews Europe* newsletter and *State of Art* report have contributed to the preparation of the Commission reports on the implementation of the *Commission Communication* and *Council Recommendation*, currently underway. This report from the Commission will help determine what has been achieved to date and the measures to be taken in the future in the field of rare diseases at European level.

### Promoting cooperation across Europe and beyond

The EUCERD was also charged with assisting the Commission in international cooperation on matters relating to rare diseases. To this end, the EUCERD has welcomed representatives of third countries, such as Japan, to its meetings to partake in discussions and share experiences. The EUCERD has also promoted liaison with other groups implicated in the field of rare diseases (e.g. IRDiRC [14], EUnetHTA [21], European Partnership Action Against Cancer [22], PARENT Joint Action [23] by inviting representatives to their meetings and including them in expert workshops, as well as participating in their meetings and workshops, to ensure that relevant experience is shared across different groups and fields. The EUCERD Joint Action is also working hard to liaise with other EC-funded projects implicated in the field of rare diseases to ensure that results are disseminated and duplication of efforts are avoided.

## Conclusion

The unique forum of stakeholders provided by the EUCERD has been a key factor in the success of the Committee in advancing discussions on important topics for the rare disease community. The Members have shown dedication and perseverance over the past three years, helping the Committee to achieve much in a relatively short space of time. Great advances have been made in developing concepts, reaching consensus and establishing recommendations for a number of key topics which will be at the centre of national and European rare disease policy in the coming years. The successful collaboration and outputs of the EUCERD is a source of inspiration for countries and regions throughout the world. The EUCERD has laid down the foundations, but much more work is needed in the future as we move from the stage of elaboration to the stage of implementation of these initiatives and policies. It is hoped that the European Commission Expert Group on Rare Diseases, which replaces the EUCERD as of January 2014, will be as efficient and productive as the former Committee in their new mandate so as to not lose the momentum gathered under the EUCERD concerning the main questions and problems faced by the rare disease community.

## Competing interests

The authors declare that they have no competing interests.

## Authors’ contributions

SA and CR carried out the analysis of the actions of the EUCERD described in this manuscript, and CR drafted and finalised the manuscript. All authors read and approved the final manuscript.
